# Activity Level Assessment Using a Smart Cushion for People with a Sedentary Lifestyle

**DOI:** 10.3390/s17102269

**Published:** 2017-10-03

**Authors:** Congcong Ma, Wenfeng Li, Raffaele Gravina, Jingjing Cao, Qimeng Li, Giancarlo Fortino

**Affiliations:** 1 School of Logistics Engineering, Wuhan University of Technology, Wuhan 430070, China; macc@whut.edu.cn (C.M.); bettycao@whut.edu.cn (J.C.); 2 Department of Informatics, Modeling, Electronics and Systems, University of Calabria, 87036 Rende, Italy; rgravina@dimes.unical.it (R.G.); liqimeng@msn.com (Q.L.); g.fortino@unical.it (G.F.)

**Keywords:** activity recognition, activity level assessment, smart cushion, body posture analysis model, activity assessment index

## Abstract

As a sedentary lifestyle leads to numerous health problems, it is important to keep constant motivation for a more active lifestyle. A large majority of the worldwide population, such as office workers, long journey vehicle drivers and wheelchair users, spends several hours every day in sedentary activities. The postures that sedentary lifestyle users assume during daily activities hide valuable information that can reveal their wellness and general health condition. Aiming at mining such underlying information, we developed a cushion-based system to assess their activity levels and recognize the activity from the information hidden in sitting postures. By placing the smart cushion on the chair, we can monitor users’ postures and body swings, using the sensors deployed in the cushion. Specifically, we construct a body posture analysis model to recognize sitting behaviors. In addition, we provided a smart cushion that effectively combine pressure and inertial sensors. Finally, we propose a method to assess the activity levels based on the evaluation of the activity assessment index (AAI) in time sliding windows. Activity level assessment can be used to provide statistical results in a defined period and deliver recommendation exercise to the users. For practical implications and actual significance of results, we selected wheelchair users among the participants to our experiments. Features in terms of standard deviation and approximate entropy were compared to recognize the activities and activity levels. The results showed that, using the novel designed smart cushion and the standard deviation features, we are able to achieve an accuracy of (>89%) for activity recognition and (>98%) for activity level recognition.

## 1. Introduction

Since sedentary behavior may cause diverse and severe health and wellness problems, adequate moderate or vigorous activity can reduce the incidence of chronic diseases, non-communicable disease and obesity. A large majority of worldwide population, such as office workers, long journey vehicle drivers, and wheelchair users spends several hours every day in sedentary activities. Many of our daily life tasks such as desk work, watching TV, eating, or commuting to work, lead us to engage in prolonged sitting activities. Therefore, monitoring sitting physical activity and assessing physical activity level is important, and can better instruct the user to engage in an active lifestyle.

In the context of Body Area Networks (BANs) [1], multi sensor fusion [2], wireless communication and embedded systems [3], many wearable devices [4] have been developed to enhance our daily lifestyle. As a consequence, several user-centric applications [5] such as smart home [6], smart chair [7], disease prediction [8] and emotion communication system [9] were developed.

To investigate the activity level of people with sedentary lifestyle, we developed a smart cushion that is able to monitor sitting postures non invasively. Activity can be recognized and activity levels can be quantified so to provide users with reminders to exercise or take a break and therefore to minimize the health risk as well as to give the users timely necessary interventions.

Our aim is to develop an effective method to achieve high accuracy for continuous activity level assessment for long-term sitting users. The main contributions of the paper are the following:

- a novel Body Posture Analysis Model (BPAM), proposed to analyze the sitting behavior;
- a novel smart cushion, with an integrated embedded unit that better combines pressure and inertial sensors;
- a method of activity level assessment, proposed to recognize activities and activity levels.

The remainder of the paper is organized as follows: Section 2 introduces the state-of-art activity level assessment method and smart cushion system; Section 3 describes the body posture analysis model, circuit design of the cushion and sensors deployment on real chair; Section 4 describes the architecture of activity level assessment. Section 5 discusses the experiment protocols and obtained results. Finally, in Section 6 some conclusions and future works are drawn.

## 2. Related Work

Posture/activity recognition detection has been a hot topic in many research areas, such as pervasive and mobile computing, context-aware computing, and ambient assisted living. The information hidden behind the posture/activity is also very meaningful to reflect the user’s health status. As pressure sensor based smart cushion is unobtrusive, several related works adopted it to recognize physical activity [10], to measure comfort and wellness [11], posture and fatigue of users sitting on normal chairs and wheelchairs [12,13]. In the following paragraph, we introduce the related work on activity level assessment, sitting posture analyze and cushion based systems.

### 2.1. Activity Level Assessment

Many studies on activity level assessment are based on accelerometers placed on the user body or use internal sensors of the smartphone. Other studies fuse heterogeneous sensor data, such as motion data from accelerometers and gyroscopes with physiological data such as electrocardiography (ECG), and electromyography (EMG). We classify the related literature in two main approaches: single sensor-based methods and multi sensor fusion methods.

#### 2.1.1. Single Sensor-Based Method

Liu et al. [14] presented a motion pattern analysis for physical activity recognition and activity level assessment by using a tri-axial accelerometer mounted on the right front waist. Their results have achieved 94.7% in accuracy, and 87.0% for three levels of activity estimation (Light, Moderate and Vigorous). He et al. [15] used a smartphone to continuously monitor user’s physical activity, detects sedentary patterns and provides personalized exercise recommendations based on user’s previous activity intensity history. Fahim et al. [16] utilized the sensors inside the smartphone, using time and frequency domain features of the accelerometer to analyze the lifestyle patterns. The raw data were transferred to a cloud server and results of activity levels were computed on the cloud platform. Liu et al. [17] proposed a method using a smartphone as a sensing and prompting device, and implement fuzzy logic prompting system to estimates exercise, including walking and running. The accumulated *activity effective index* is used to analyze exercise performance based on pattern recognition in the activity effective analysis stage.

#### 2.1.2. Multi Sensor Fusion Method

Grillon et al. [18] proposed a method using two inertial sensors attached on the wheelchair’s bottom and left wheel to detect accelerometer and gyroscope data plus a smartwatch to detect accelerometer and heart rate data; several activities with different intensity levels of wheelchair users were recognized. Duclos et al. [19] combined the use of two complementary devices: smartphone and smartwatch to collect the accelerometer data to detect sedentary activities (sitting, reclining posture), movements (walking) and periods of more intense body movements (running). With the feedback results, the proposed system can promote users’ self-management and daily efficiency evaluation of physical activity. Liu et al. [20] presented a sensor fusion method using accelerometer (body motion) and ventilation sensor (breathing) to identify physical activity and the corresponding energy expenditure. Jung et al. [21] developed an inspection service middleware by analyzing health parameters such as electroencephalography (EEG), electrocardiography (ECG), oxygen saturation (SpO2), blood pressure (BP), and respiration rate (RR). Activity assessment model based on Expectation Maximization (EM) algorithm was used to assess users mental wellness and recommend them proper activity.

### 2.2. Sitting Posture Analysis and Smart Cushion Systems

During sitting conditions, the trunk, pelvis and thigh are mostly determinant of the posture. Mork et al. [22] investigated sitting posture and low back muscle activity for female computer workers. Data collected from pelvis, upper trunk, and left thigh were used to determine back posture and identify periods with sitting condition. Scena et al. [23] analyzed the variation in the characteristics of pelvis and trunk displacements during sitting posture especially for the wheelchair user in order to help them preventing pressure sores. Also Geffen et al. [24] decomposed the seating posture with trunk, pelvis and thighs, and developed a chair controlled by body postures. Maradei et al. [25] simulated the driving tasks and investigated the movements of pelvis and trunk; low back pain was examined by the frequencies of macro repositioning movements in prolonged sitting posture.

As cushion based posture recognition system is noninvasive and easy to implement, many researchers choose this technology to recognize the postures/activities. There exist two main production technologies of smart cushions for motion detection: the first one is based on a pressure sensor array such as e-textile, whereas the second one relies on the use of fewer individual sensors deployed on the seat or backrest. Compared with using sensor arrays, recent research focused on using fewer, independent sensors with advantage of low cost and easier marketing promotion.

Yu et al. [26] proposed PoSeat, a smart cushion equipped with an accelerometer under the seat and pressure sensors both on the seat and backrest. A hybrid SVM classifier was used to recognize and monitor the postures in order to prevent chronic back pain. Barba et al. [27] used 16 pressure sensors (8 on the seat and 8 on the backrest) connected to an Arduino board to develop an on-line posture recognition system to monitor users’ affective states, such as boredom, attention and nervousness, during learning scenarios. Zemp et al. [28] developed an instrumented chair with force and acceleration sensors to identify the user’s sitting postures using machine learning methods. Sixteen force sensor values and the backrest angle (determined by the accelerometer fixed on the backrest) were used as the features for the classification. Cheng et al. [29] investigated user activity detected from simple pressure sensors mounted under the legs of a chair. Results showed that it is possible to detect not only different postures, but also subtle hand and head related actions like typing and nodding. Fu et al. [30] proposed a robust, low-cost, sensor based system that is capable of recognizing sitting postures and activities. Eight force sensing resistors (FSRs) were placed on chair backrest and seat, and a Hidden Markov Model approach was used to establish the activity model from sitting posture sequences. Kumar et al. [31] have designed Care-Chair with just 4 pressure sensors on the backrest of a chair. Equipped with intelligent data analytics, their system can classify 19 kinds of complex user sedentary activities and it can also detect user functional activities and emotion based activities. Similarly to the smart cushion, Zhu [32] designed a low-cost device using Arduino and pressure sensor unit to quantify postural stability. Approximate entropy features of Center-of-Pressure (CoP) were used to reflect the irregularity of oscillations. Our former research [33] used 3 pressure sensors (two of which on the seat and the third one on the backrest) and an accelerometer attached on the waist to detect user’s postures sitting on a smart wheelchair. Different kinds of body swing movement can be recognized and experimental results showed high classification accuracy.

To better summarize the related work on cushion based systems, in Table 1 we reported the features and classification method that they used and also the accuracy they obtained.

Smart cushion based systems are noninvasive and can uncover valuable knowledge from the postures. To the best of our knowledge, a smart cushion with an integrated unit to better fuse accelerometer and pressure sensors signal has never been proposed before. In the following, we will discuss our novel smart cushion and the analysis method adopted in our activity level assessment system.

## 3. Measurement Method

In the following paragraphs, we describe the body posture analysis model of sitting behavior, explain our novel smart cushion and the deployment schema of the sensors on a real chair.

### 3.1. Body Posture Analyze Model of Sitting Behavior

**Figure 1:**
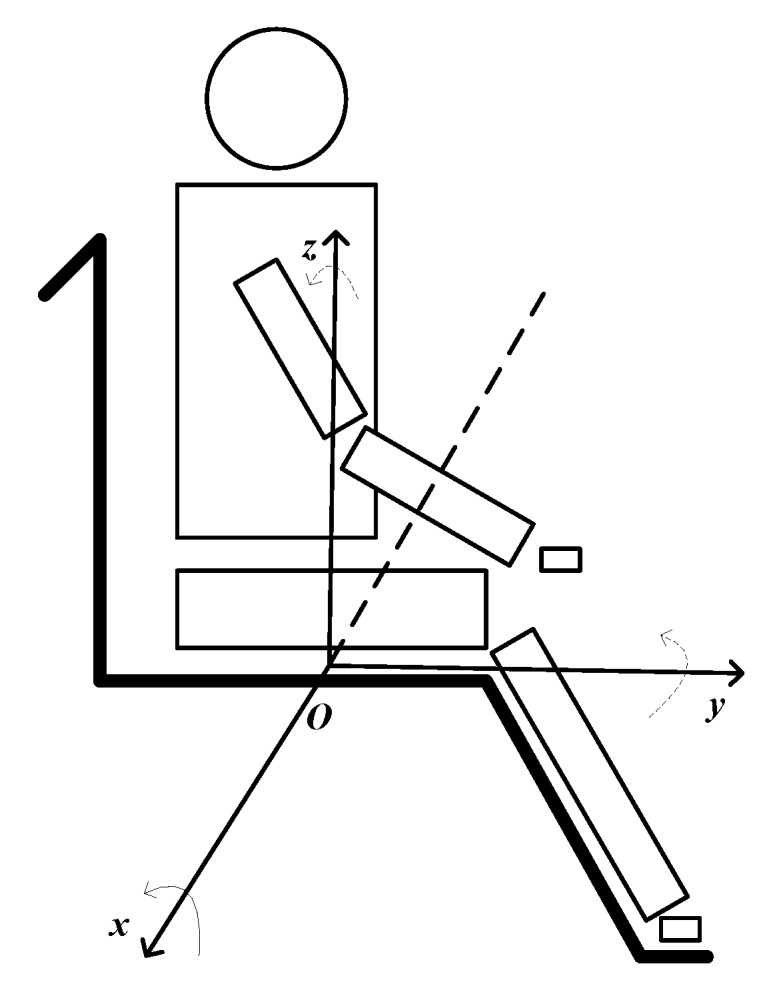
Body posture analyze model of sitting behavior.

Based on former studies [22,23,24,25] of sitting posture analysis, we construct the Body Posture Analysis Model (BPAM) of sitting behavior considering these three main body parts of trunk, pelvis and thigh, as shown in Figure 1. We defined the front and back sides of the sensors with respect to the body location as the *x* axis, left-right as the *y* axis, and up-down as the *z* axis. The *x* axis reflects the body swing from anterior-posterior direction, and the *y* axis reflects the body swing from medial-lateral direction. The variation of rotation angles velocity can better provide the extent of body swing.

### 3.2. The Novel Designed Smart Cushion

**Figure 2:**
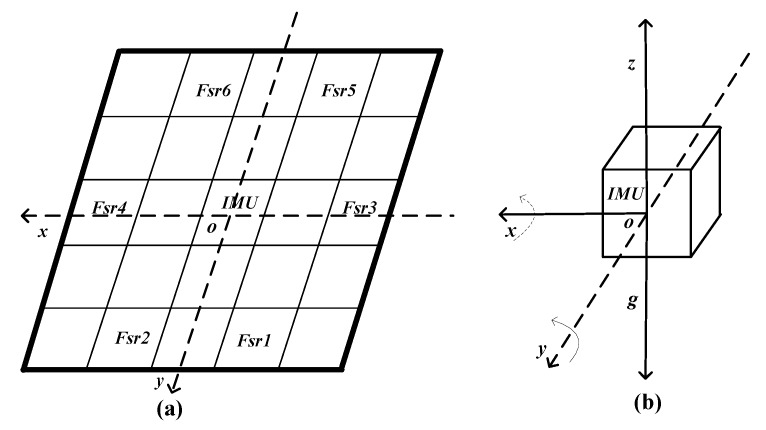
Sensors deployment of the smart cushion: (**a**) schematic diagram of the sensors deployment on the base board; (**b**) IMU sensor and the three axis representation

Two types of sensors are used in our cushion: six pressure sensors and a 9-axis inertial measurement unit. The deployment of the sensors on the cushion is shown in Figure 2.

Compared with the previous research on smart cushion design that use pressure sensors and accelerometers [26,28,33] as shown in Table 1, our novel cushion shows three advantages:

- it is one integration unit that better combine the pressure sensors and accelerometer;
- it is easy to deploy, since all the sensors were deployed on the cushion seat;
- it is noninvasive, with the facility to monitor the sitting postures and activities.

#### 3.2.1. Pressure Detection Unit

With respect to our former research [34,35], we designed the cushion with pressure sensors’ deployment as shown in Figure 2a. The pressure sensor used in this work is the FSR406 [36] that is produced by Interlink Electronics. As force is applied on the sensing areas, the resistance value of the pressure sensor will be correspondingly altered. It is robust against mechanical stress and easy to deploy on any surface. The force sensitivity is in the range of (0.1, 10) kg, and the pressure sensitivity is in the range of (1.5, 150) kg/cm${}^{2}$. Assuming a conventional size for the seat of 40 cm × 40 cm, we split it into 5 × 5 square zones. The six pressure sensors are individually placed in different zones. Specifically, the two pressure sensors Fsr3 and Fsr4 have been deployed to the left and right sides of the seat to better deal with individual physiognomy differences.

The displacement of the Center of Pressure (CoP) can assess postural stability in the field of biomechanics [32]. The CoP is defined as the point location of the vertical seat reaction force vector. It represents a weighted average of all the pressures over the surface area in contact with the seat. In our case, six pressure sensors were placed in definite pressure points.

As in Figure 2, the central point is assumed as original point *O*, and each Fsr sensor has its own location. In order to simplify the calculation, we use the relative distance from each sensor to the original point. Here the coordinate of each sensor can be represented as shown in Table 2.

And then the CoP value for the two axis can be calculated as follows:

(1) $$\begin{array}{c} CoP_{x}=\frac{\sum f_{i}*x_{i}}{\sum f_{i}} \end{array}$$

(2) $$\begin{array}{c} CoP_{y}=\frac{\sum f_{i}*y_{i}}{\sum f_{i}} \end{array}$$

where $f_{i}$ is the pressure readings of each sensor, and $\left(x_{i},y_{i}\right)$ represents the coordinates of the sensor, with i∈ {1,2,3,4,5,6}.

#### 3.2.2. Inertial Measurement Unit (IMU)

MPU9250 is a 9-DoF IMU [37], placed in the center of the cushion, on the opposite side with respect to the pressure sensors, as shown in Figure 2b. It consists of two chips: the MPU-6500 (which contains a 3-axis gyroscope, a 3-axis accelerometer, and an onboard Digital Motion Processor (DMP) capable of processing complex Motion Fusion algorithms) and the AK8963 (a 3-axis digital compass). In this work we only use the 3-axis gyroscope to detect the anterior-posterior and medial-lateral swings extent. The IMU provides angular velocity $Gyro_{x}$ and $Gyro_{y}$ of the two axises.

### 3.3. Implementation of Smart Cushion

**Figure 3:**
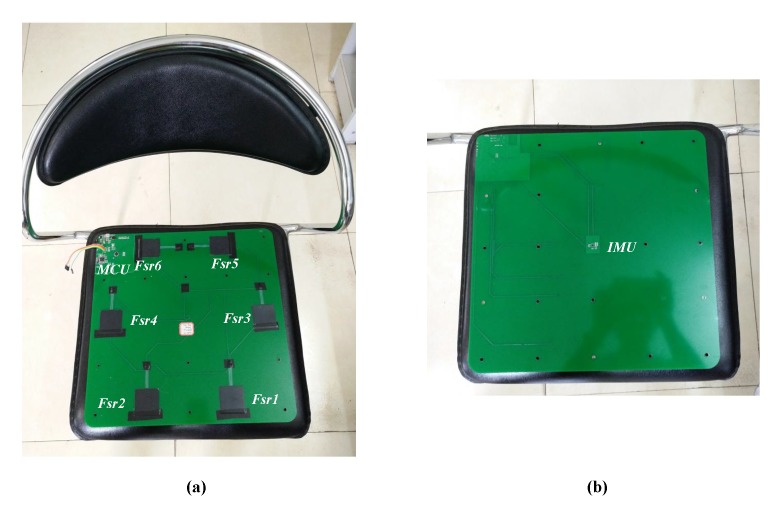
Deployment of the smart cushion circuit board on a real chair: (**a**) front of the circuit board; (**b**) back of the circuit board.

The circuit board of the smart cushion is shown Figure 3. Figure 3a shows the top side of circuit board. Six pressure sensors were deployed as described before.

On the left-top of the circuit board is located the control circuit unit, that is composed of the following components:

- Micro Controller Unit (MCU), to process the sensor data using Arduino Pro Mini [38];
- Bluetooth communication unit, to send the processed results to remote devices (such as computer, smartphone, or tablet);
- Vibration Motor, controlled by the MCU, is used to alarm the users;
- Power supply circuit, to provide the power to the circuit board.

Figure 3b shows the opposite side of the circuit board; the IMU is placed in the center of the board. The circuit board will be eventually embedded in the foam filling of the cushion for user comfort.

## 4. Activity Level Assessment Method

**Figure 4:**
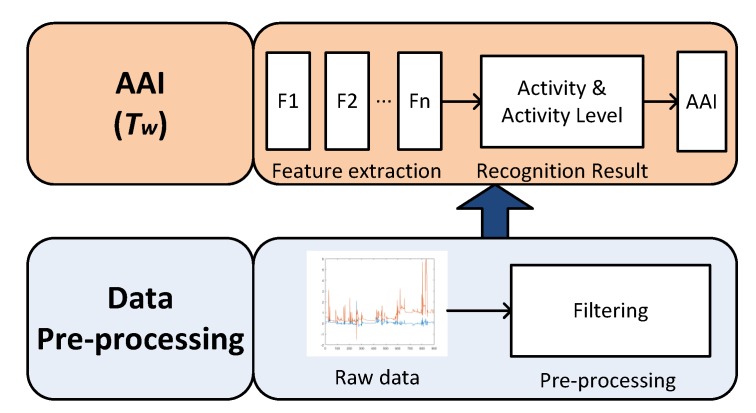
Activity level assessment workflow.

Using the smart cushion described in the former paragraph, we can assess activity levels of users sitting on the chair. The system architecture is shown in Figure 4. It is composed of two layers: data pre-processing and activity assessment index calculation.

### 4.1. Data Pre-Processing

Using the smart cushion, we are able to collect the time series of sampled data. Instance vectors are generated from the pressure sensors and IMU. In order to capture small movements of body, data was collected at a frequency of 10 Hz. After the wave filtering period, at the time *t*, we can get the raw data vector $P_{t}=\left(CoP_{x,t},CoP_{y,t},Gyro_{x,t},Gyro_{y,t}\right)$.

### 4.2. Activity Assessment Index

Using the raw data collected as described in Section 4.1, we can extract significant features. In this work we selected two kinds of feature sets, one is using standard deviation and the other is using approximate entropy (ApEn). Both standard deviation and ApEn values are calculated over the time series data. We set the time slide window as $T_{w}$(s), with the overlap of 50% to determine each feature.

Standard deviation is a measure that is used to quantify the amount of variation or dispersion of a set of data values. A low standard deviation indicates that the data points tend to be close to the mean (also called the expected value) of the set, while a high standard deviation indicates that the data points are spread out over a wider range of values. Using Matlab, we can easily calculate the standard deviation value. The feature vector can be represented as $F_{std}=\left(Std_{cx},Std_{cy},Std_{gx},Std_{gy}\right)$.

ApEn is a technique used to evaluate the regularity and unpredictability of fluctuations over time-series data. As to a chosen data segment, ApEn determines the probability similarity of the next set of segments in the same duration; the higher the probability the smaller the ApEn value, indicating less complexity of the data. A time series containing many repetitive patterns has a relatively small ApEn; a less predictable (i.e., more complex) process has a higher ApEn. ApEn is usually a real number between 0 and 2. Theoretically, a perfectly repeatable time series data would yield ApEn value closed to 0, and the perfectly random time series data would yield closed to 2.

ApEn was first proposed by Pincus [39] and it is widely used to evaluate the complexity of the data. Its popularity stems from the fact that it can be applied to both short- and long-term data recordings. Also Pincus demonstrated that ApEn can calculate the complexity within relatively short data sets (even as 75 to 100 data points). This is a great advantage that involves a serial of applications, such as fatigue detection and activity level assessment, as this rank index is increased as time accumulated.

Given a sequence of series data $S_{N}$ {u(1), u(2), ..., u(N)} and *m* an integer representing the length of compared run of data, we form a sequence of m-dimensional vectors x(1), x(2), ..., x(N−m+1). Now, x(i) = {u(i), u(i+1), ..., u(N−m+1)}, for each *i*, 1≤i≤N−m+1. Then, we calculate the distance between x(i) and x(j); notice that x(j) takes all other vectors. d[x(i),x(j)] represents the distance between x(i) and x(j). Also, we need to determine the threshold *r* (r>0).

The ApEn formula can be finally expressed as follows:

(3) $$\begin{array}{c} C_{i}^{m}\left(r\right)=\frac{\left(number\;of\;j\;such\;that\;d[x(i),x(j)]<=r\right)}{N-m+1} \end{array}$$

(4) $$\begin{array}{c} C^{m}\left(r\right)={\left(N-m+1\right)}^{-1}\sum_{i=1}^{N-m+1}log\left(C_{i}^{m}\left(r\right)\right) \end{array}$$

(5) $$\begin{array}{c} ApEn\left(N,m,r\right)=ln\left[\frac{C^{m}\left(r\right)}{C^{m+1}\left(r\right)}\right] \end{array}$$

To calculate each ApEn value, there exist three parameters: *N*, *m* and *r*. In the former paragraph we introduced that Pincus [39] demonstrate that the value *N* can be set as a relative short data set (even as 75 to 100 data point), then *N* can be calculated as $N=10\times t_{w}$. The parameter m=2 is set to provide reasonable results in most clinical data research [40]. The typical value of *r* is 0.2 multiplied by the standard deviation of the data samples in a time slide window; it is called tolerance threshold for accepting similar patterns between the neighboring segments [40]. Using the ApEn formula, we can obtain the feature vector $F_{apen}=\left(ApEn_{cx},ApEn_{cy},ApEn_{gx},ApEn_{gy}\right)$.

After the feature extraction, we can feed the features to the classifier to recognize the activities and activity levels. As our aim is to implement the system on embedded devices, the recognition phase must be computationally lightweight. With the similar problem of posture detection in [35], decision tree is chosen also in this work as the suitable classifier to the recognition of postures.

## 5. Experiments and Results

In this section, we describe the experiment protocol, time window selection for the sedentary activity recognition and the recognition results of using 10-fold and Leave One Out (LOO) Cross Validation (CV). For practical implications and actual significance of results, we selected wheelchair users among the participants to our experiments.

### 5.1. Experiment Protocol

Wheelchair-bound population suffers of prolonged exposure to high pressure of the buttock, which restricts blood flow and leads to tissue necrosis, which in turn might cause pressure ulcers [41]. So it is useful to encourage the wheelchair users to migrate sedentary activity to some extent body movements.

Different activities and activity level/intensity were summarized in Table 3. The table also reports examples of activities that can fall under a specific activity level performed by the wheelchair users, defined as follows:

- Light intensity activity level,
- Moderate intensity activity level, and
- Vigorous intensity activity level.

Our principle of defining the three kinds of activity levels is following the table in [42]. Physical activity level can be measured by using the Metabolic Equivalent of Task (MET) [43] which is a physiological measure expressing the energy cost of physical activities. Originally, 1 MET was considered as the Resting Metabolic Rate (RMR) obtained during quiet sitting, while MET values of activities range from 0.9 (sleeping) to 23 (running at 22.5 km/h). It is estimated the light intensity activity level is at the range of (1, 3) METs, moderate intensity activity level is at the range of (3, 6) METs, and vigorous intensity activity level is (6, 9) METs. A MET value greater than 9 indicates the user is in very vigorous activity. Reading, desk working and conversation could fall into the light intensity activity level [42]. The swing activity that the wheelchair user needs to be prompted to do could fall into the moderate intensity activity level. The activity of doing exercise like lifting a weight might instead fall into the vigorous intensity activity level.

Experiments were conducted during two weeks and have been carried out in our laboratory; we recruited 8 persons (5 males and 3 females). All the participants were informed of the purpose and procedure of the study and, after signing an informed consent, they filled a form including questions on gender, ethnicity, age, height, and weight. Participants has the tolerance of sitting on the wheelchair for 2 h, with no active pelvis or thigh injury, with no history of cardiovascular disease. Their ages were in the range of [60, 65], all of them are in healthy status and with no limb injury. They can stand a little, but most of the time they need a wheelchair to facilitate their moving demands. Their BMI is in the range of [16, 34], whose distribution in our experiment sample is shown in Table 4.

During the experiment, they were free to choose the time period for the test session and they were asked to perform common activities on wheelchair among (1) Reading a book; (2) Desk working at a computer; (3) Having a conversation (in peace status); (4) Swing left-right or front-back; (5) Doing physical exercise such as lifting a weight. On average, experiment sessions lasted about 2 h and each participant had to perform 6 sessions over the two weeks. All the experiment sessions have been video recorded so to manually label the samples of each performed activity with one of the three defined activity levels.

### 5.2. Data Processing and Time Window Choosing

In order to test our designed cushion and system, we analyzed the data using WEKA data mining toolbox [44]. A 10-fold cross-validation using J48 [45] has been adopted to realize the classifier [35].

J48 is a specific decision tree implementation of the well known C4.5 algorithm using the concept of information entropy. The classifier model is generated by a training procedure that uses a set of pre-classified samples. Each sample is a *p*-dimensional vector also known as a feature vector. Each node of the tree represents a decision (typically a comparison against a threshold value); at each node, J48 chooses the feature (i.e., an attribute) of the data that most effectively splits its set of samples into distinct subsets according to the normalized information gain (NIG). In particular, the algorithm chooses the feature with the highest NIG to generate the decision node. The main parameters used to tune the classifier generation are the pruning confidence (C = 0.25) and the minimum number of instances per leaf (M = 2).

Using the experiment protocol described in Section 5.1, we can get the activity database. We selected about 50 min data for each activity. As for activity recognition and activity level recognition, researchers usually choose the time slide window in the range of [2, 30] s, or even reach to 430 s (for the activity of walking or fast walking). Sampling frequency and time window set in previous studies are summarized in Table 5.

**Figure 5:**
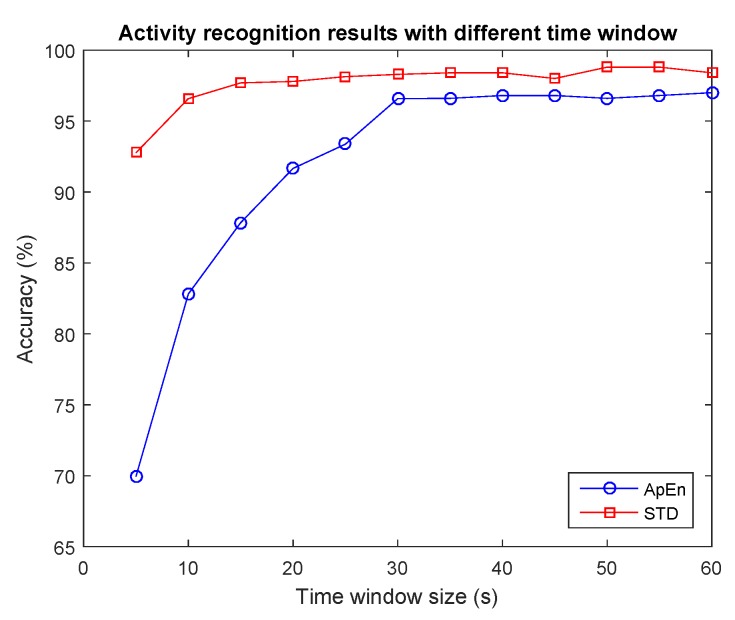
Activity recognition results with different time slide window.

It has been shown that small window size is effective to detect physical activity on devices with reduced resources [17]. However, with sedentary activities like reading a book or desk working, relatively longer time window are usually required. In order to determine the time window more rationally for activity recognition, we examine the time slide window in the range of [5, 60] s, with an epoch size of 5 s. The recognition results of using the two kinds of features is shown in Figure 5; we can see that with larger window size the recognition results will increase. With a compromise of computation and recognition accuracy, 30 s is an optimal option for our designed system.

Referring to previous literature as shown in Table 5, we particularly examine the effect of using time slide window 10 s, 20 s, 30 s separately. F-measure is a measure of a test’s accuracy. It considers both the precision (*p*) and the recall (*r*) of the test. *p* is the number of correct positive results divided by the number of all positive results, and *r* is the number of correct positive results divided by the number of positive results. F-measure can be interpreted as a weighted average of the precision and recall, where F-measure reaches its best value at 1 and worst at 0. Here we examine the F-measure value for the recognition of each kind of activity; results are reported in Table 6. The table shows that for short time slide windows (10 and 20 s), with sedentary activity, the F-measure value is lower. It can be noted that better result of F-measure can be achieved using a time window of 30 s.

**Figure 6:**
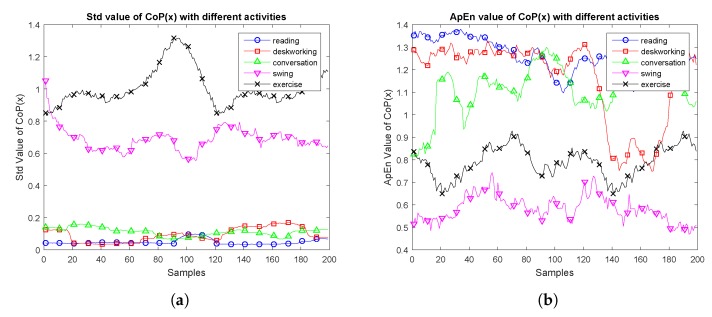

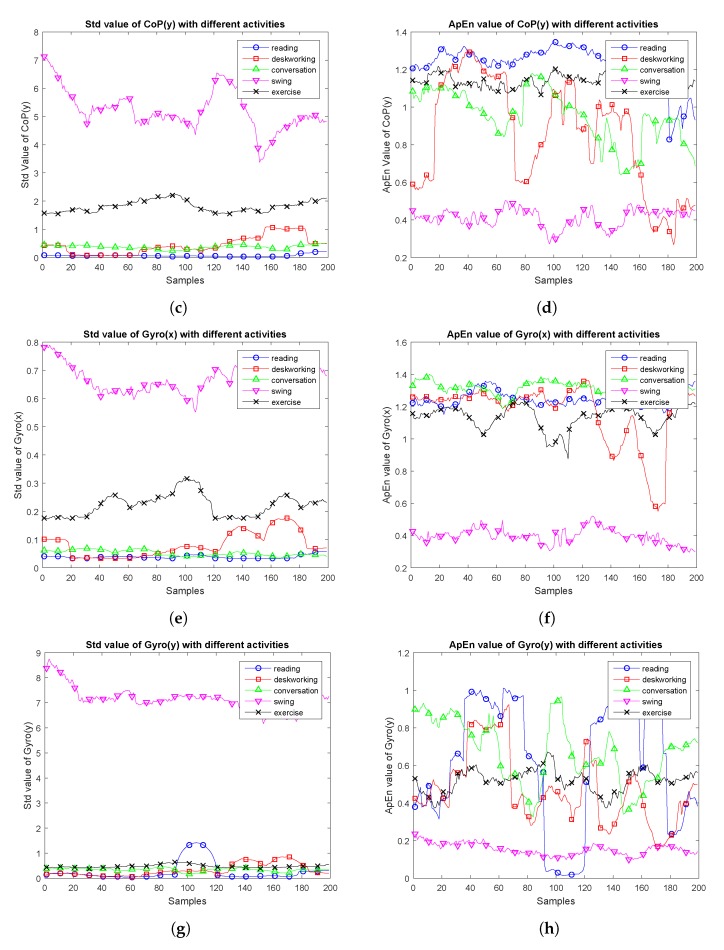
Feature distribution of each activity.

As the time slide window is set at 30 s and the sampling frequency is set to 10 Hz, each time slide window contains 300 raw data, with 50% overlap. To give an exemplification, the plots in Figure 6 showed the features in terms of Std and ApEn. The *x*-axis represents the number of samples. The *y*-axis represents the Std and ApEn values.

As shown in Figure 6, the Std value of the four kinds of data with the three kinds of low-intensity level activities (reading, desk working and conversation) are relatively lower than the other two kinds of activities. ApEn features do not show clear difference like Std features, as the ApEn reflects the complexity of the sequence sample data.

### 5.3. Results Comparison with k-Fold Cross Validation

**Figure 7:**
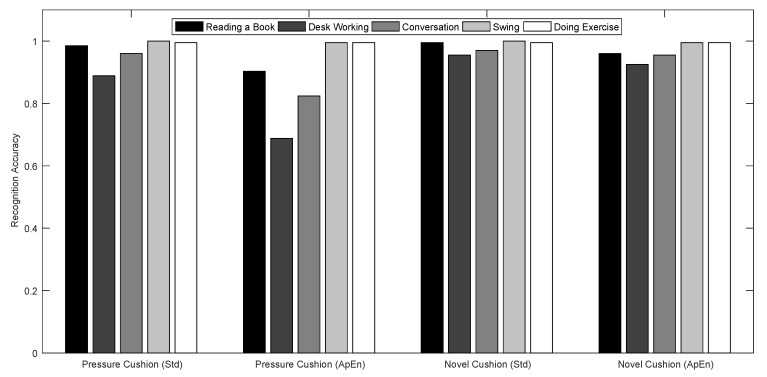
Recognition results for different activities.

**Figure 8:**
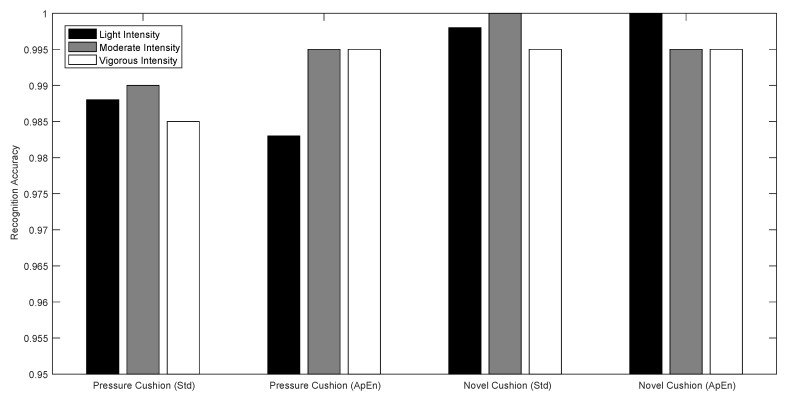
Activity level recognition for different activities.

The recognition results for each activity and activity level using 10-fold cross validation are depicted in Figure 7 and Figure 8.

As shown in Figure 7, compared with using the pressure cushion, the novel designed cushion accuracy exceeds 90% either for using Std features or ApEn features. Also, it can be noted that the recognition of Swing and Doing Exercise activities can achieve higher accuracy, as these two kinds of activities are performed in a regular pattern. Using the novel cushion, the recognition results of the other three kinds of sedentary activities can be improved. This shows that our novel designed cushion has the advantage of being able to monitor small body movements.

As clearly shown in Figure 8, compared with using the pressure cushion, the novel designed cushion have better performance. As to the activity level recognition, both cushion and features has the higher recognition accuracy of more than 98%.

#### 5.3.1. Recognition Results Using Pressure Sensors Unit

The recognition results achieved using only the pressure sensors unit are shown in Table 7 and Table 8. As to the recognition of different activities, using Std features higher accuracy can be achieved than using ApEn features. In particular, with ApEn features, for the recognition results of three low level intensity activities, lower accuracy can be observed. The two kinds of features provide similar results for the recognition of activity levels; it would be preferable to choose the Std feature due to its quicker computation time. Using both ApEn and Std features, we would obtain similar results as only using Std features.

#### 5.3.2. Recognition Results Using the Novel Designed Cushion

Recognition results obtained using the novel designed cushion are shown in Table 9 and Table 10. Similarly to the results with only the pressure sensor unit, the Std features show better performance than ApEn features. Adding the features extracted from $Gyro_{x}$ and $Gyro_{y}$ to the features extracted from the pressure signals, the activity recognition results are improved. As can be noted in Table 10, using the novel cushion both Std features and ApEn features can lead to higher activity level recognition results. Using both ApEn and Std features, we would obtain similar results as only using Std features. This means that it is enough to only use the Std features for the recognition of activities and activity levels.

### 5.4. Results Comparison with Leave One Out Cross Validation

In Section 5.3, we evaluate the system using 10-fold cross validation. We found that using our novel designed cushion and the features of standard deviation is an optimal solution of recognizing activities and activity levels. In order to decrease the over-fitting of the sample data, we evaluate the system using Leave One Out (LOO) Cross Validation (CV). It is usually used involving human subjects to account for the subject-to-subject variation that occurs and also for the tendency to autocorrelation for time series data involving a single subject. LOO is probably the best method to estimate the risk when learning a model, whereas 10-fold CV is more accurate for model selection [46].

**Figure 9:**
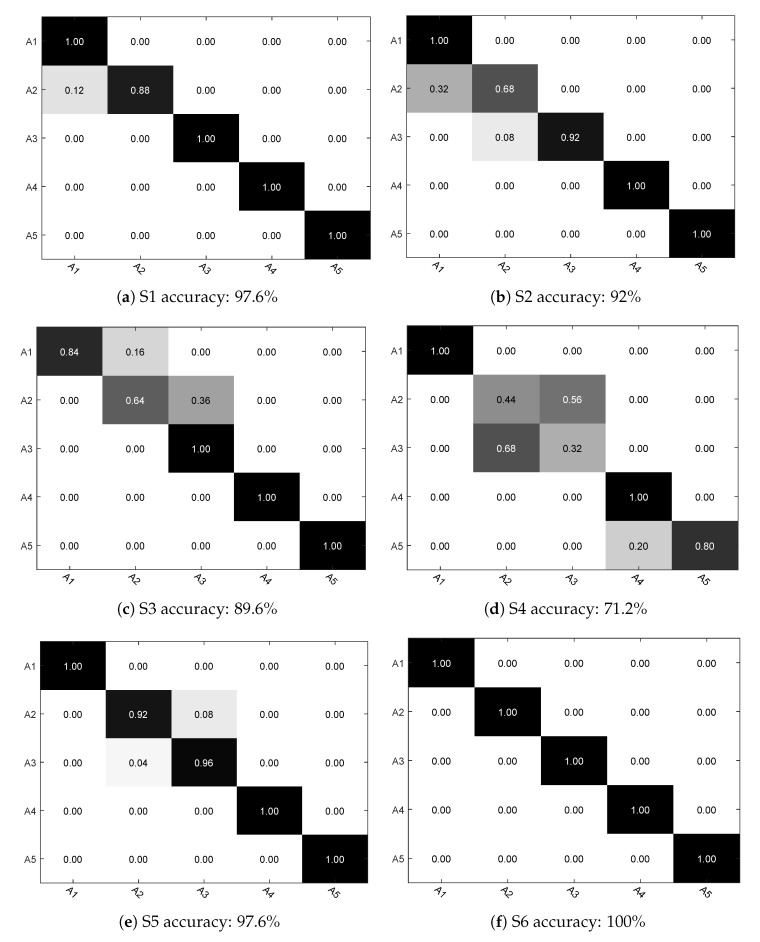

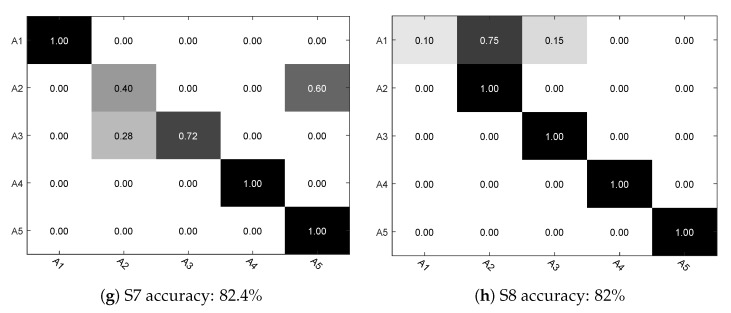
Confusion matrix of predicted activities for each subject.

As shown in Figure 9, we give the activity recognition results using LOO cross validation for each subject (S1–S8). Here we use A1, A2, A3, A4 and A5 to represent the five kinds of activities. Overall, the average recognition results is 89.05%. As we can see, for the swing activity and doing exercise, in most of the cases, excellent accuracy can be achieved. However, for the recognition of reading a book, desk working and conversation, the accuracy is not satisfactory as these activities present similar features values and are therefore not easy to distinguish.

**Figure 10:**
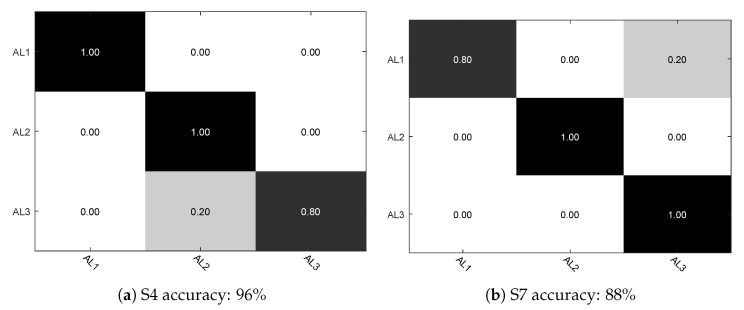
Confusion matrix of predicted activity levels of subject S4 and S7.

As shown in Figure 10, we give the activity level recognition results using LOO cross validation for each subject. Here we use AL1, AL2 and AL3 to represent the three kinds of activity levels. With subjects S1, S2, S3, S5, S6, S8 the obtained accuracy is 100%, so it is not presented in Figure 10. The overall average recognition accuracy is 98%, which can definitely suit our goal of recognizing each activity level.

## 6. Conclusions

Activity recognition and level assessment of people with sedentary lifestyle is very useful and can promote physical exercise and lead to more active life. In this paper, we proposed a method of activity classification and activity level assessment using a novel designed smart cushion. The cushion combines the pressure sensors and IMU better than previous systems. It is suitable for monitoring the sitting behavior in contexts such as workplace, car, or on the wheelchairs and can be easily implemented with low-cost embedded devices. Experiments on wheelchair users have been carried out; activity recognition and activity level assessment have been performed.

Future works will be devoted to recognizing more kinds of sitting activity and to design a mechanism for a finer grained activity level assessment, also we will recruit more subjects and collect more data in order to provider a robust, reliable results. Furthermore, we will include the monitoring of other body parts such as upper and lower limb in order to provide a total body activity analysis model. Finally, we plan to investigate the integration of physiological sensor data [47,48] to get more comprehensive health information and provide a quantitative metric of activity level, specifically to recognize abnormal high intensive activities for the detection of user physical and mental discomfort.

## Figures and Tables

**Table 1:** Related works on cushion based systems.

Author	Pressure Sensor	Accelerometer	Integrate	Features	Classification	Accuracy
			Unit		Method	
Yu et al. [26]	2 on the seat and	Backrest	No	N/A	SVM	N/A
	4 on the backrest					
Barba et al. [27]	8 on the seat and	No	-	N/A	N/A	N/A
	8 on the backrest					
Zemp et al. [28]	16 pressure sensors	Backrest	No	N/A	SVM, MNR,	90.9%
					Boosting,	
					NNs, RF	
Cheng et al. [29]	4 under the	No	-	Mean, RMS,	LDA	88%
	chair leg			Center of weight		
Fu et al. [30]	4 on the seat and	No	-	N/A	HMM	N/A
	4 on the backrest					
Kumar et al. [31]	4 on the backrest	No	-	Mean and	ERT	86%
				variance, FFT etc.		
Zhu [32]	4 pressure sensors	No	-	Approximate Entropy	N/A	N/A
Ma et al. [33]	2 on the seat and	Waist	No	Mean and standard	J48	96.85%
	1 on the backrest			deviation		

SVM: Support Vector Machine; MNR: Multinomial Regression; NNs: Neural Networks; RF: Random Forest; HMM: Hidden Markov Model; ERT: Extremely Randomized Trees.

**Table 2:** The coordinate of each pressure sensor.

Sensor No.	Coordinate (*x*, *y*)
*Fsr*1	−1, 2
*Fsr*2	1, 2
*Fsr*3	−2, 0
*Fsr*4	2, 0
*Fsr*5	−1, −2
*Fsr*6	1, −2

**Table 3:** Categorization of different activity levels of wheelchair users.

Activity Level	Description	Activities
Light intensity	User performs common daily life activities in sitting condition.	Reading a book, Desk working, Conversation
Moderate intensity	User performs moderate activities to prevent pressure ulcer.	Swing left-right or front-back
Vigorous intensity	User is doing exercise to keep fit.	Doing exercise

**Table 4:** BMI distribution of the subjects participating to the experiments.

Description	Underweight	Normal	Overweight and Obese
BMI	<18.5	[18.5, 25)	≥25
Number of subjects	2	4	2

**Table 5:** Sampling frequency and time window chosen in literature.

Research	Sampling Frequency (Hz)	Time Window (s)
Liu, K. et al. [14]	40	10, 270, 430
Liu, C. et al. [17]	40	2
Liu, S. et al. [20]	30	30
Zhu, Y. et al. [32]	10	30

**Table 6:** F-measure for each activity with different time window.

	10 s		20 s		30 s	
	*ApEn*	*Std*	*ApEn*	*Std*	*ApEn*	*Std*
Reading	0.78	0.962	0.88	0.98	0.967	0.988
Desk Working	0.665	0.922	0.812	0.949	0.92	0.962
Conversation	0.776	0.953	0.902	0.967	0.95	0.972
Swing	0.985	0.997	0.995	0.997	0.997	0.997
Doing Exercise	0.828	0.995	0.992	0.995	0.995	0.995
Mean F-measure	0.828	0.966	0.916	0.978	0.966	0.983

**Table 7:** Activity recognition for using the pressure sensor cushion.

	Std Feature			ApEn Feature			Std & ApEn Feature		
	Accuracy	Precision	F-Measure	Accuracy	Precision	F-Measure	Accuracy	Precision	F-Measure
Reading	0.985	0.975	0.98	0.93	0.845	0.885	0.98	0.97	0.975
Desk Working	0.889	0.947	0.917	0.688	0.815	0.747	0.879	0.926	0.902
Conversation	0.96	0.918	0.939	0.824	0.808	0.816	0.945	0.913	0.928
Swing	1.0	0.995	0.997	0.995	1.0	0.997	1.0	0.995	0.997
Doing Exercise	0.995	0.995	0.995	0.995	0.957	0.975	1.0	1.0	1.0
Total Results	0.966	0.966	0.966	0.886	0.885	0.884	0.961	0.961	0.961

**Table 8:** Activity level recognition for using the pressure sensor cushion.

	Std Feature			ApEn Feature			Std & ApEn Feature		
	Accuracy	Precision	F-Measure	Accuracy	Precision	F-Measure	Accuracy	Precision	F-Measure
Light intensity	0.998	1.0	0.999	0.983	0.998	0.991	0.998	1.0	0.999
Moderate intensity	1.0	0.995	0.997	0.995	0.995	0.995	1.0	0.995	0.997
Vigorous intensity	0.995	0.995	0.995	0.995	0.952	0.973	1.0	1.0	1.0
Total Results	0.998	0.998	0.998	0.988	0.988	0.988	0.999	0.999	0.999

**Table 9:** Activity recognition for using the novel designed cushion.

	Std Feature			ApEn Feature			Std & ApEn Feature		
	Accuracy	Precision	F-Measure	Accuracy	Precision	F-Measure	Accuracy	Precision	F-Measure
Reading	0.995	0.98	0.988	0.96	0.974	0.967	0.995	0.98	0.988
Desk Working	0.955	0.969	0.962	0.925	0.915	0.92	0.95	0.974	0.962
Conversation	0.97	0.975	0.972	0.955	0.945	0.95	0.98	0.975	0.977
Swing	1.0	0.995	0.997	0.995	1.0	0.997	1.0	0.995	0.997
Doing Exercise	0.995	0.995	0.995	0.995	0.995	0.995	1.0	1.0	1.0
Total Results	0.983	0.983	0.983	0.966	0.966	0.966	0.985	0.985	0.985

**Table 10:** Activity level recognition for using the novel designed cushion.

	Std Feature			ApEn Feature			Std & ApEn Feature		
	Accuracy	Precision	F-Measure	Accuracy	Precision	F-Measure	Accuracy	Precision	F-Measure
Light intensity	0.998	1.0	0.999	1.0	0.997	0.998	0.998	1.0	0.999
Moderate intensity	1.0	0.995	0.997	0.995	1.0	0.997	1.0	0.99	0.995
Vigorous intensity	0.995	0.995	0.995	0.995	1.0	0.997	0.995	1.0	0.997
Total Results	0.998	0.998	0.998	0.998	0.998	0.998	0.998	0.998	0.998
